# Subjective measures of disability in children and adolescents: opportunities, challenges, and implications

**DOI:** 10.3389/fresc.2025.1531740

**Published:** 2025-02-11

**Authors:** Shahram Moradi, Christian Møller-Skau

**Affiliations:** ^1^ Faculty of Health and Social Sciences, Department of Health, Social and Welfare Studies, University of South-Eastern Norway, Porsgrunn, Norway; ^2^ Research Group for Health Promotion in Settings, Department of Health, Social and Welfare Studies, University of South-Eastern Norway, Tønsberg, Norway

**Keywords:** self-reports, disability, children, adolescents, subjective measure

## Introduction

The assessment of disability among children and adolescents in research has traditionally relied on objective measures, such as clinical evaluations and standardized tests or proxy evaluation (1–3). However, there is a growing recognition of the importance of subjective measures in line with the United Nations Convention on the Rights of Persons with Disabilities (4) definition—those that capture the young individual's personal experiences and perceptions of their disabilities. The UNCRPD advocates for a transition from traditional medical models to a social and human rights-based framework, ensuring that disability assessments prioritize the lived experiences and rights of individuals with disabilities, rather than focusing solely on individual impairments (5, 6). By highlighting the significance of participation and inclusion, the UNCRPD underscores the value of subjective measures in evaluating the extent to which these objectives are achieved among children and adolescents with disabilities [see the works by (7, 8)]. This opinion article explores the implications of using subjective measures of disability, with a special focus on children and adolescents, highlighting their potential benefits, challenges, and scenarios in which subjective disability measures may not be suitable.

Self-report and subjective measures are often used interchangeably but have distinct characteristics and applications in research and clinical assessment. In the context of disability, self-report measures often involve individuals providing information about their own experiences of disability, typically through standardized questionnaires or interviews (9–11). This could include questions about physical functioning, difficulties with mobility, or the impact of disability on daily life. Since self-reports rely on an individual's personal perspective, they capture a subjective view of disability that may vary widely across respondents due to differences in perception, coping mechanisms, or self-awareness (12–14). Today, the Washington Group on Disability (15) are well-known examples of measures for self-reporting disability among children and adolescents (16). The standardized tools from the WG are widely used to measure functional limitations across different domains, such as seeing, hearing, walking, cognition and mental health, among diverse populations such as older children and adolescents (17). The set of questions is particularly valuable in survey contexts, providing comparable data on disability prevalence, which helps inform policy decisions, guide interventions, and promote inclusivity (15). According to a report by Massey et al. (18), parents and teenagers generally agree on observable impairments, such as issues with movement, vision, and hearing, as indicated by the questionnaires from the WG. However, they often have differing views on less visible problems, like anxiety. The report suggests that self-assessment can be beneficial in these situations, but parents can still provide valuable insights into their teen's functional abilities.

Subjective measures of disability, however, are broader in scope and can include a range of assessment methods beyond self-reports. For example, subjective assessments might integrate observations and evaluations from caregivers or healthcare professionals, as well as insights from family who are familiar with the individual's daily life (19). These assessments could also include semi-structured interviews or narrative approaches that allow for a more nuanced understanding of how disability is experienced beyond a questionnaire's constraints (20). The Pediatric Evaluation of Disability Inventory (PEDI), which is a subjective clinical assessment tool designed to assess functional capabilities and performance in children with disabilities, ages 6 months to 7.5 years (21). Administered by clinicians like occupational or physical therapists, the PEDI relies heavily on input from parents or caregivers who are familiar with the child's abilities.

### The merits and limitations of subjective measures and self-reports

Subjective measures of disability offer distinct advantages over objective measures by capturing personal experiences and addressing unobservable aspects of disability that objective tests may fail to consider (13, 22, 23). Examples of such tools include the Pediatric Evaluation of Disability Inventory–Patient Reported Outcome (PEDI-PRO) (24) and the Child and Adolescent Scale of Participation (CASP), which assess activities and participation among adolescents (25). In addition, subjective measures are also sensitive to psychosocial factors, addressing aspects such as social relationships, and emotional well-being, as demonstrated by instruments like the Functional Disability Inventory (FDI) (26). Moreover, subjective measures and self-reports, such as the WG-SS, can be adapted to different cultural contexts and conditions, making them valuable for international research and diverse populations (27).

Despite their benefits, subjective measures and self-reports also present limitations and challenges. One significant concern is the potential for bias in them (28, 29), which can stem from factors such as age-related differences in cognitive abilities, self-awareness and communication skills, especially among younger children. Eddy et al. (30) highlight the importance of understanding how children interpret items in self-report measures. Their study revealed that many children answered questions despite not fully understanding them, underscoring a significant gap in effective communication and comprehension within pediatric populations. In addition, younger children may struggle to accurately express their experiences, while fluctuating emotional states or situational factors may also compromise reliability (31). Shulz et al. (3) revealed the limitations of the WG-SS in identifying psychosocial disabilities among adolescents and younger adults. Chi and Lin (32) suggest that the PEDI may not fully capture the real-life performance of daily living activities in preschool children with autism spectrum disorder (ASD). Their study underscores the importance of incorporating observational assessments alongside self-report measures.

A practical example can illustrate the advantages and limitations of subjective measures. Consider a school-based assessment program for adolescents with mobility disabilities. The program used the CASP to evaluate participation in school activities. While the adolescents' self-reported data highlighted reduced participation due to inaccessible infrastructure, caregivers and teachers reported that psychosocial factors, such as peer relationships and self-confidence, played a larger role in limiting engagement. This case demonstrates how subjective measures can capture psychosocial barriers often overlooked by objective metrics. However, it also highlights the challenges of relying solely on self-reports, such as variability in individual responses and potential gaps in self-awareness among younger participants.

Cultural differences can also impact how disability is perceived and reported, potentially skewing results if assessments are not culturally sensitive (33, 34). For example, Gannotti et al. (33) showed that differences exist between Puerto Ricans and the norms established in the United States for the performance of functional skills by children. The study further reported that in Puerto Rico, cultural values such as interdependence and overprotectiveness shape parental expectations and perceptions of children's capabilities, which can affect the interpretation of disability assessments like the PEDI.

### Amplifying marginalized voices of children and adolescents with disabilities using subjective measures and self-reports

The development of subjective measures and self-reports of disability for children and adolescents provides a critical opportunity to amplify the voices of marginalized groups within this population (e.g., children and adolescents with intellectual disabilities, those with multiple disabilities, ethnic minorities with disabilities, and youth from low-income or rural areas) [see the study by (35) for example]. We reason that, unlike objective measures, which often fail to capture the nuanced and lived experiences of marginalized groups, subjective measures and self-reports empower children and adolescents to share their unique challenges and perspectives. This approach is essential for ensuring that the voices of these groups are not overlooked and that their needs are adequately represented in research, policy, and practice.

However, there is a significant lack of knowledge and research regarding the effective development and application of these tools for marginalized groups. Existing literature often overlooks how systemic barriers, such as discrimination and limited access to resources in schools (36), which we believe that can impact on the accuracy, reliability, and inclusivity of self-reported data. This gap underscores the urgent need for studies that explore how subjective measures can better capture the diverse experiences of all children and adolescents with disabilities, particularly those from marginalized backgrounds.

### Enhancing the usability of subjective measures and self-reports

Several strategies can be employed to enhance the usability of subjective measures and self-reports for children and adolescents. Age-appropriate language and formats should be used to ensure questions are easily understood and accessible to younger populations (37). Coombes et al. (38), in their systematic literature review on enhancing self-report health outcome measures for children, concluded that developing patient-reported outcome measures for children and young people requires careful design due to their varying developmental stages and cognitive abilities. In addition, authors also concluded that children under 5 years cannot reliably report health outcomes, necessitating the use of proxy measures. For 8-year-olds, recall should be limited to 48 h, and only dichotomous response formats are reliable. Further, children prefer visually appealing, computerized formats for self-reporting health outcomes, suggesting that incorporating these features can improve the acceptability and completion rates of outcome measures among young people. We propose that for adolescents, the use of digital tools alongside adaptive designs—such as language tailored for age-appropriate comprehension and avoidance of complex terminology—can significantly enhance the usability of self-reports (30).

We suggest creating a supportive environment during assessments can help children feel comfortable expressing their experiences, thereby reducing social desirability bias. This idea aligns with Article 12 of the United Nations Convention on the Rights of the Child, which affirms children's right to express their views (39). Involving children in the development of these measures ensures that their perspectives and cultural contexts are considered, enhancing the relevance and accuracy of the assessments (30, 40). Knox and Usen (41) showed that the accuracy of self-reported disability measures in children and adolescents can be improved through structured interviews with knowledgeable caregivers, as used in the PEDI, and by combining standardized assessments with self-reports.

### When not to use self-report measures

We reason that self-report measures may be inappropriate for younger children or individuals with cognitive impairments who may struggle to understand questions or provide reliable responses (38). In contexts where variability in self-reports complicates comparisons across populations or when evaluating intervention effectiveness, combining self-report with objective measures may provide a more comprehensive evaluation.

### Research gaps and future directions

Despite the growing recognition of the importance of subjective measures, several research gaps remain. Longitudinal studies are needed to assess how subjective experiences of disability evolve over time, particularly as children transition into adolescence and adulthood. There is also a lack of comprehensive research on the intersectionality of disability with social determinants such as socioeconomic status, gender, and cultural background, which can impact how disabilities are experienced and reported. Furthermore, developing culturally sensitive subjective measures that reflect diverse experiences is crucial. Integrating technology in subjective assessments, such as mobile applications or online platforms, can facilitate real-time self-reporting and provide immediate feedback enhancing data collection and offering a more comprehensive view of children's experiences. Collaborative efforts between researchers, practitioners, and policymakers are essential to translate findings from subjective assessments into actionable interventions and policies that promote inclusivity and support for children and adolescents with disabilities.

## Conclusion

While self-report measures empower young individuals by giving them a voice in their assessments, challenges related to bias, reliability, and cultural sensitivity that must be addressed. It is imperative for researchers and practitioners to adopt a balanced approach that integrates both subjective and objective assessments, fostering a more comprehensive understanding of disability. Identifying research gaps highlight the need for longitudinal studies and the development of culturally sensitive measures. The integration of technology in subjective assessments offers innovative solutions for real-time data collection and feedback. Addressing these challenges and pursuing future research directions will improve outcomes for children and adolescents with disabilities, fostering a more inclusive environment and enhancing well-being.
